# Elevated limb-bud and heart development (LBH) expression indicates poor prognosis and promotes gastric cancer cell proliferation and invasion via upregulating Integrin/FAK/Akt pathway

**DOI:** 10.7717/peerj.6885

**Published:** 2019-05-06

**Authors:** Ruoxi Yu, Zhi Li, Chuang Zhang, Huicong Song, Mingming Deng, Liping Sun, Ling Xu, Xiaofang Che, Xuejun Hu, Xiujuan Qu, Yunpeng Liu, Ye Zhang

**Affiliations:** 1 Department of Medical Oncology, the First Affiliated Hospital of China Medical University, Shenyang, China; 2 Key Laboratory of Anticancer Drugs and Biotherapy, Shenyang, China; 3 Department of Respiratory and Infectious Disease of Geriatrics, the First Affiliated Hospital of China Medical University, Shenyang, China; 4 Tumor Etiology and Screening Department of Cancer Institute and General Surgery, the First Affiliated Hospital of China Medical University, Shenyang, China

**Keywords:** LBH, Gastric cancer, Prognositic biomarker, TCGA, Integrin

## Abstract

The limb-bud and heart development (LBH) gene is a highly conserved, tissue-specific transcription cofactor in vertebrates that regulates multiple key genes in embryonic development. The role of LBH in various cancer types is still controversial, and its specific role and molecular mechanism in the oncogenesis of gastric cancer (GC) remains largely unexplored. In the present study, the prognostic significance and clinicopathological characteristics of LBH in GC was determined. The LBH mRNA expression was first investigated in four independent public datasets (TCGA-STAD, GSE15459, GSE29272, and GSE62254) and then validated with our samples at the protein level. LBH was overexpressed at both the mRNA and protein levels in cancer compared with normal tissues. High LBH expression was correlated with advanced T, N, and M stages. Kaplan–Meier analysis and log-rank test indicated that higher LBH expression was statistically correlated with shorter overall survival (OS) in the public datasets and our study samples. Univariate and multivariate Cox regression analysis showed that LBH was an independent prognostic biomarker for survival in TCGA-STAD, GSE15459, GSE62254 cohorts, and our GC patients. *In vitro* experiments showed that knockdown of LBH can significantly inhibit the proliferation and invasion of HGC-27 cells, while overexpression of LBH can significantly enhance the proliferation and invasion of BGC-823 cells. Gene Set Enrichment Analysis (GSEA), Gene ontology (GO) and Kyoto Encyclopedia of Genes and Genomics (KEGG) indicated that high LBH expression is associated with the PI3K-Akt pathway, focal adhesion, and extracellular matrix (ECM)-receptor interaction. Western blot analysis showed that knockdown of LBH significantly inhibited the expression of integrin α5, integrin β1, p-FAK, and p-Akt. Therefore, results from the present study indicate that LBH is a potential independent prognostic biomarker and promotes proliferation and invasion of GC cells by activating the integrin/FAK/Akt pathway.

## Introduction

Gastric cancer (GC) is the most common and lethal type of malignancy worldwide (Katona & Rustgi, 2017). In 2012, approximately 989,600 new cases of GC and 738,000 cases of GC-related deaths were recorded worldwide, accounting for 6.7% and 8.8% of new cancer cases and cancer deaths, respectively (Torre et al., 2015). Despite conventional postoperative adjuvant therapy, approximately 1/3 of patients still relapse (Bang et al., 2012). The poor prognosis is due to the lack of clear preventive measures, early detection methods, and effective treatment for GC. Therefore, identifying useful clinical biomarkers and molecular targets related to GC progression is necessary.

The limb-bud and heart development (LBH) gene is a highly conserved, tissue-specific transcription cofactor in vertebrates that regulates multiple key genes in embryonic development (Al-Ali et al., 2010; Briegel & Joyner, 2001). The gene is located on chromosome 2p23.1 and encodes a small nuclear protein with 105 amino acids. In addition to embryonic tissues, LBH is expressed in adult organs, including the spleen, gut, kidney, brain, and peripheral nervous system. Aberrant expression of LBH during heart development can lead to congenital heart diseases such as partial trisomy 2p syndrome and other growth defects (Briegel et al., 2005; Briegel & Joyner, 2001; Conen et al., 2009; Ekwall et al., 2015; Li et al., 2015; Powder et al., 2014). Increasing evidence indicates that embryonic development and tumorigenesis have similar molecular mechanisms (Kahn, 2014). Rieger et al. first discovered the role of LBH in cancer in 2010 (Rieger et al., 2010). They observed that LBH acts as a target gene for the Wnt pathway to inhibit mammary epithelial differentiation and promote Wnt-induced tumorigenesis (Lindley et al., 2015). Since then, LBH has received attention as a new tumor-associated gene, and its role in nasopharyngeal carcinoma (Liu et al., 2015), hepatocellular carcinoma (Chen et al., 2018), prostate cancer (Liu et al., 2018), and lung adenocarcinoma (Deng et al., 2018) has been studied. In a study by Liu, overexpression of LBH was shown to lead to G1/S phase arrest in nasopharyngeal carcinoma and act as a transcriptional cofactor of NF-κB (Liu et al., 2015). Results from studies by Chen have confirmed that LBH predicted poor prognosis in hepatocellular carcinoma (Chen et al., 2018). Moreover, our team found that LBH is downregulated and predicts better overall survival (OS) outcome in lung adenocarcinoma (Deng et al., 2018). However, the expression and biological functions of LBH in GC remain unclear.

Therefore, in this study, the expression pattern of LBH in GC was examined in public datasets and in our study samples. In addition, the effects of LBH on cell proliferation, migration, and invasion of GC cells was explored. The potential mechanism of LBH was also investigated using bioinformatics and western blot analyses.

## Materials & Methods

### Analysis of LBH expression in TCGA and GEO data sets

RNASeqV2 level3 data (STAD) of 375 GC patient samples with complete clinical data were downloaded from TCGA data portal (http://cancergenome.nih.gov/). Microarray data set GSE15459, GSE29272 and GSE62254 were downloaded from the Gene Expression Omnibus (GEO) database. GSE29272 contains 134 pairs of cancerous tissues and paired normal tissues. GSE15459 and GSE62254 contains 192 and 300 GC patient samples with complete clinical data, respectively.

To analyze the association of LBH expression with survival data, we first dichotomized the samples in each dataset to two groups, denoted as LBH-high and LBH-low, by its median expression level of their respective dataset. Difference of the overall survival rate between the two groups is tested by log-rank test with *P* < 0.05 as the significance cutoff.

To validate the association of overall survival with LBH expression and other clinical features, univariate Cox regression model were used to estimate Hazard Ratio (HR) and 95% confidence interval (CI). To test the independence of the association between LBH and overall survival, multivariate Cox model was further constructed based on the Akaike information criterion (AIC) value using “forward” stepwise selection methods.

### Patients and samples

This study was approved by the Human Ethics Review Committee of the First Affiliated Hospital of China Medical University (AF-SOP-07-1.1-01). GC tissues and corresponding normal tissues were collected from 82 patients who underwent gastrectomy between 14 April 2014 and 25 May 2017 in the Oncology Institution, First Affiliated Hospital of China Medical University, Shenyang, China. The standard requirements for patients included in the study were: (1) Histologically confirmed GC; (2) No history of other malignancy or other severe diseases that may influence the outcome of our follow-up; (3) No prior neoadjuvant chemotherapy. Demographic and clinical characteristics such as gender, age, initial diagnosis date, and tumor stage at the time of initial diagnosis were obtained from medical records and pathology reports. Follow-up is performed every six months, and the follow-up time is defined as the date from the pathological diagnosis to the date of death or the date of the last follow-up. This study follows the Helsinki Declaration.

### Immunohistochemistry (IHC) staining

Formalin-fixed paraffin-embedded tissues from primary gastric cancer were cut into 4 µm thick sections for immunohistochemical staining. Immunohistochemical staining was performed using the streptavidin-peroxidase method. The sections were deparaffinized, hydrated, and soaked in 3% H_2_O_2_ for 10 min at room temperature, and then incubated with LBH polyclonal antibody (1:1,000, ab122223, Abcam) overnight at 4 °C. At the same time, the negative control and non-immune rabbit IgG were incubated with the same dilution as the primary antibody. On the second day, the specimen was washed with PBS and then incubated with biotinylated secondary antibody for 10 min at room temperature. The specimens were then stained with diaminobenzidine (DAB) and counterstained with 20% hematoxylin. Immunohistochemistry reagents were purchased from Maixin Biotechnology (Fuzhou, China).

### Immunostaining evaluation

The assessment was performed independently by two investigators in multiple region of the same sample blinded to clinical data. The extent of LBH staining was scored by a semi-quantitative method that rates the staining intensity (SI) and percentage of positively stained cells (PP) to derive immunoreactive scores (IRS), IRS = PP × SI. The SI was defined as follows: 0 points for no staining, 1 point for weak coloring (light yellow), 2 points for moderate coloring (yellow), and 3 points for strong coloring (brown). The PP of LBH tumor cells was scored on a scale of 0 to 4 (0, 0%–9% positive tumor cells; 1, 10%–25% positive tumor cells; 2, 26%–50% positive tumor cells; 3, 51%–75% positive tumor cells; 4, >75% positive tumor cells). Three high-power fields (200 ×) were selected for each tissue. Patient sample were divided into high expression group and low expression group based on IRS. High expression of LBH is defined as a moderated and strong staining.

### Gene sets enriched by high level of LBH using GSEA

Gene Set Enrichment Analysis (GSEA) is conducted by using GSEA v2.2.2 (http://www.broadinstitute.org/gsea) to identify LBH associated gene sets (Subramanian et al., 2005). LBH expression is first dichotomized as low and high categories to annotate phenotypes. GSEA of genes’ correlations with the phenotypes is further tested by using C2: CP KEGG gene sets from MSigDB (Li et al., 2016). The gene sets that are significantly enriched by the genes associated with high expression of LBH [false discovery rate(FDR) < 0.05] were selected as enriched gene sets.

### Gene ontology (GO) and Kyoto Encyclopedia of Genes and Genomes (KEGG) analyses

To determine how LBH affects the prognosis of gastric cancer patients, we performed Gene ontology (GO) and Kyoto Encyclopedia of Genes and Genomes (KEGG) analysis of LBH co-expressed genes. The LBH co-expressed genes were calculated by R, sorted according to the Spearman correlation coefficient, and the top 1000 genes were sorted for the next GO and KEGG analysis. These gene functional enrichment analyses were performed using the clusterProfiler package of R. When the *P*.adj value is less than 0.05, the GO term or the KEGG pathway was identified as being significantly enriched by these genes. The GOplot package of R software was used to demonstrate the results of the GO and KEGG analyses.

### Cell line

The gastric cell lines BGC-823, HGC-27, MKN-45 and SGC-7901 were purchased from the Academy of Military Medical Science (Beijing, China). The cells were cultured in RPMI-1640 containing 10% fetal bovine serum (FBS) at 37 °C in a humidified atmosphere of 5% CO_2_.

### Reverse-transcription-polymerase chain reaction (RT-PCR)

Total RNA was isolated was referring to our previous method (Xu et al., 2017). RT-PCR was performed with primer pairs for LBH: forward (5′-CCTGAGGAGTTCCTGGTCC-3′) and reverse (5′-CAGATGCTGGCTGGTATGAC-3′). For 18S as control: forward (5′-GGTGAAGGTCGGAGTCAACGG-3′) and reverse (5′-GAGGTCAATGAAGGGGTCATTG-3′). PCR conditions were 50 °C for 2 min, 95 °C for 10 min; 45 cycles of 95 °C for 15 s, 60 °C for 1 min, 72; one cycle of 95 °C for 15 s, 60 °C for 1 min, 95 °C for 30 s, 60 °C for 15 s.

### Western blot

Cells were extracted and protein was quantified as described previously (Zhang et al., 2015b). The cells were washed twice with phosphate-buffered saline (PBS), lysed in lysis buffer (1% Triton X-100, 50 mM Tris-HCl pH 7.4, 150 mM NaCl, 10 mM EDTA, 100 mM NaF, 1 mM Na3VO4, 1 mM PMSF, 2 µg/ml aprotinin) and quantified using the BCA protein quantification kit (cat. no. ab102536; Abcam). The cell lysates were separated by 8% or 15% SDS-PAGE, the samples were transferred to a nitrocellulose membrane (Immoblin-P, Millipore; Merck KGaA). After blocking with 5% skim milk in tris-buffered saline Tween-20 (TBST) buffer (10 mM Tris-HCl pH 7.4, 150 mM NaCl, 0.1% Tween-20) at room temperature for 1 h, antibodies were added and incubated overnight at 4 °C. Following three washes with TBST buffer, the membrane was incubated with secondary goat anti-rabbit and goat anti-mouse antibodies for 30 min at room temperature followed by three washes with TBST buffer. Finally, the protein bands were detected with enhanced chemiluminescence reagent (SuperSignal™ Western Pico Chemiluminescent Substrate; Pierce; Thermo Fisher Scientific, Inc.) and scanned using the Electrophoresis Gel Imaging Analysis System (DNR Bio-Imaging Systems, Neve Yamin, Israel).

### Lentivirus transfection

Lentiviruses for LBH overexpression or knockdown and control vector were purchased from the Genechem (Shanghai, China). The cells were cultured in 6-well plates, and after reaching 70% confluence, medium containing lentivirus and polybrene (5 µg/ml; Genechem) was added at a multiplicity of infection (MOI) of 10 and mixed with the cells. Polybrene is used to increase infection efficiency. After 24 h of incubation, the supernatant in the wells was replaced with RPMI-1640 containing FBS and cultured for 5 days. In order to establish the stable cell line, puromycin (cat. no. P7130; Sigma-Aldrich; Merck Millipore) was used as a selection marker for the infected cells. The expression efficiency was evaluated by qRT-PCR and western blot analysis.

### Colony formation assay

The cells transfected with lentiviruses for LBH or vector were trypsinized and counted. Five hundred cells were implanted in each dish. The cells were cultured for 14 days in RPMI-1640 medium containing 10% FBS, then the cells were fixed, stained and photographed.

### Cell migration (wound-healing) assay

Cells plated on six-well plates for 48 h. A confluent monolayer of cells was wounded by scratching a line with a 200 µl sterile pipette tip. The old medium is then aspirated and replaced with new medium without FBS. The wound was photographed by using an inverted microscope at time points of 0 and 24 h. The percentage of open area was analyzed and quantified by the ImageJ software. Each independent experiment was repeated at least three times.

### Transwell

Cells in logarithmic growth phase (70%–80% in integration of state) were made into cell suspension (1.0 × 10^5^/ml for migration and 2.0 × 10^5^/ml for invasion) and plated 0.1 ml per well into transwell upper chamber (Corning, NewYork, USA). For the invasion assay, the upper layer of the chamber was covered with a 1:30 dilution of Matrigel 24 h in advance. Then removed the transwell chamber after 24 h culture. The inner surfaces of cells were erased using a cotton swab dipped in serum-free medium. Membrane was immersed in 75% ethanol for 15 min and stained using the Wright-Giemsa method. Ordinary optical microscope was chosen to observe cells penetrate the membrane of the lower chamber, counting four high power field and averaging analysis. The experiment was repeated thrice.

### Statistical analysis

All statistical analyses were performed using SPSS18.0. The relationship between LBH expression and clinical pathological parameters were assessed by *χ*^2^ test. Kaplan–Meier survival curves were compared using a log-rank test. Univariate Cox regression analysis were performed on available clinical pathology parameter. Multivariate Cox-regression analysis with forward stepwise selection and an entry limit of *P* < 0.05 was performed to identify independent predictors of survival in the patient cohort. All statistical tests are two-tailed. *P* < 0.05 was considered statistically significant.

## Results

### Expression of LBH is elevated in GC in TCGA-STAD and GSE29272 cohorts

**Figure 1:**
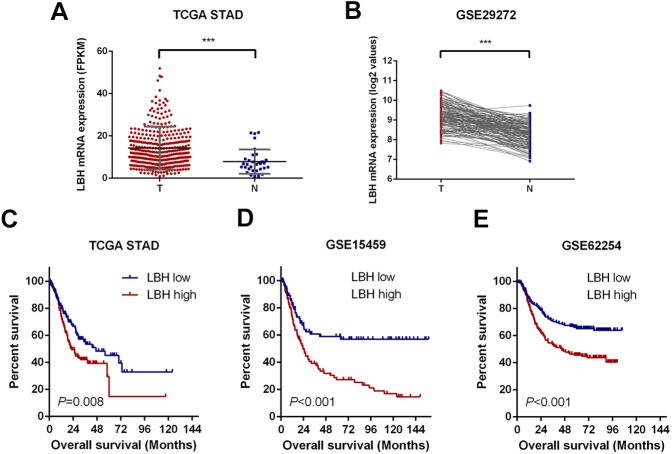
LBH is upregulated in gastric cancer tissues and correlates with poor survival. (A) Dot plots represent LBH expression levels in gastric cancer tissues (*n* = 375) and normal gastric tissues (*n* = 32) according to the data from the TCGA-STAD cohort. *P* value was determined using Student’s t-test. Error bars represent mean ± s.d., *** *P* < 0.001. (B) LBH expression in paired normal (*N* = 134) and GC tissues (*N* = 134) in GSE29272 cohort. *P* value was determined using Student’s t-test. *** *P* < 0.001. (C–E) LBH expressions were associated with overall survival in the gastric cancer patients. (C) Data was retrieved from TCGA-STAD cohort. The number of subjects in LBH high (top 50%) and low (bottom 50%) were *n* = 187 and *n* = 188. *P* = 0.008 by log-rank test. (D) Data was retrieved from the GSE15459 cohort. The number of subjects in LBH high (top 50%) and low (bottom 50%) were *n* = 96 and *n* = 96. *P* < 0.001 by log-rank test. (E) Data was retrieved from the GSE62254 cohort. The number of subjects in LBH high (top 50%) and low (bottom 50%) were *n* = 150 and *n* = 150. *P* < 0.001 by log-rank test.

First, the LBH mRNA expression levels were predicted in GC and adjacent tissues using the online datasets TCGA-STAD and GSE29272. By analyzing the LBH mRNA levels in unpaired GC (*N* = 375) and normal tissues (*N* = 32) of the TCGA-STAD cohort, LBH mRNA expression was significantly upregulated in GC tissues (*P* < 0.001, Fig. 1A). Upregulation of LBH mRNA in GC was also confirmed by comparison of paired tumors and adjacent non-tumor tissues in GSE29272 (*N* = 134, *P* < 0.001, Fig. 1B). These data indicated that LBH is upregulated in GC tissue and may contribute to GC.

### Upregulation of LBH was closely associated with poor prognosis of patients in TCGA-STAD, GSE15459, and GSE62254 datasets

To assess the prognostic value of LBH mRNA expression in GC, the relationship between LBH mRNA expression and OS was evaluated in three independent datasets with sufficient number of patients using Kaplan–Meier analysis and log-rank test. Specifically, the samples were divided into LBH high expression group and LBH low expression group based on LBH median expression level and the OS was compared between groups. A significant negative association between LBH expression and patient survival (*P* = 0.008) was observed in the TCGA-STAD cohort (Fig. 1C). Similarly, log-rank test showed that patients with low LBH expression in the GSE15459 and GSE62254 cohorts had significantly longer OS (*P* < 0.001) than patients with high LBH expression (Figs. 1D–1E). The above survival analysis indicated that high LBH expression predicted poor prognosis in GC.

### LBH expression correlated with poor clinicopathological features in the TCGA-STAD, GSE15459, and GSE62254 cohorts

**Figure 2:**
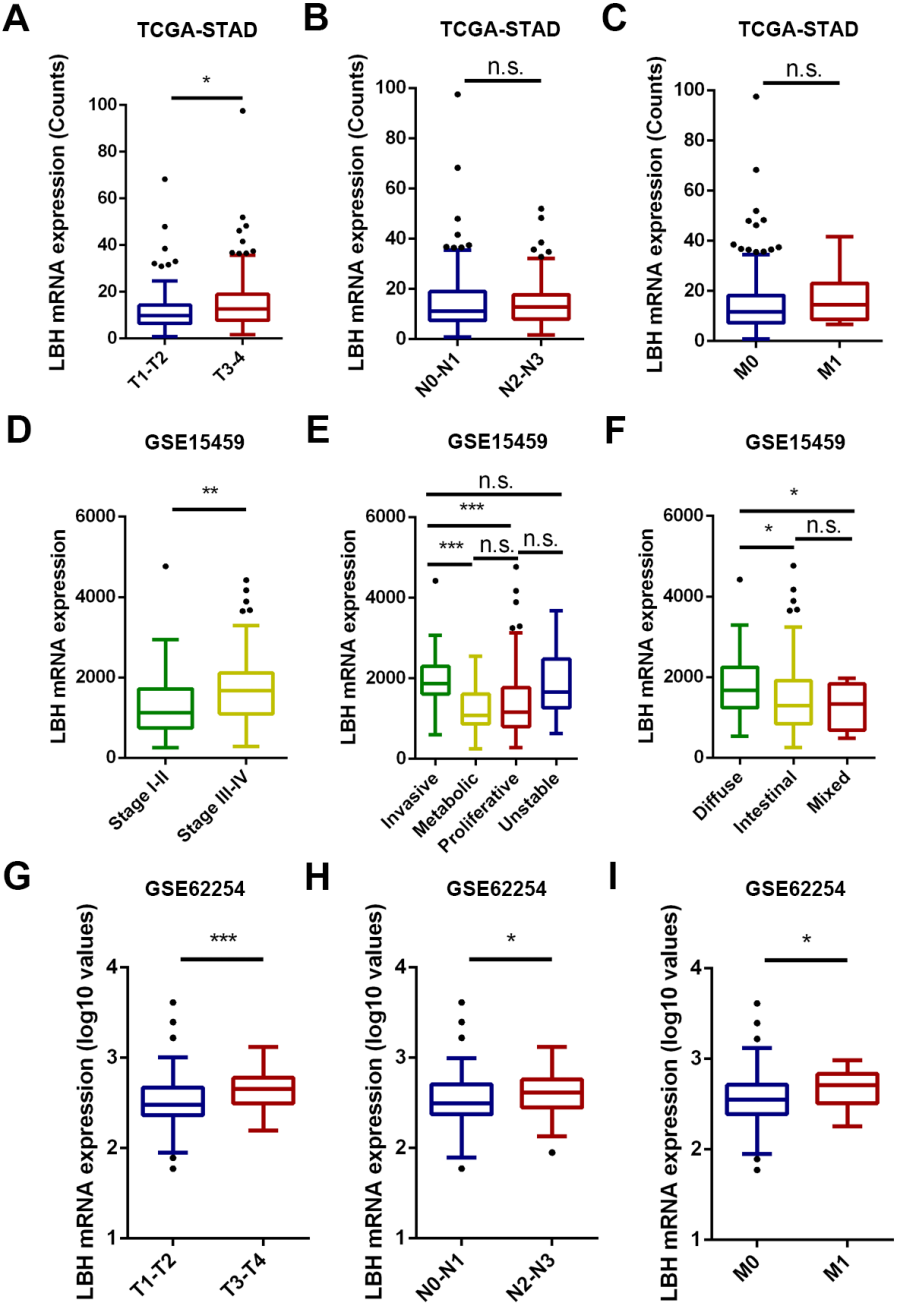
The box and whisker plot (tukey style, outliers in black dots) represent the expression patterns of LBH mRNA in GC predicted by bioinformatics. (A–C) LBH mRNA was significantly upregulated in T3-T4 than in T1-T2 sample (A). LBH mRNA was not significantly changed with N and M stage (B–C). (D–F) The LBH mRNA was dramatically more abundant in stage III-IV (left panel), invasive subtype (E) and diffused Lauren classification (F) in GSE15459 cohort. (C) The LBH increased from early T stage (G), N stage (H) and M stage (I) to the late T, N, M stage in GSE62254. *P* value was determined using Student’s t-test. * *P* < 0.05, ** *P* < 0.01, *** *P* < 0.001, n.s., not significant.

The association between the LBH mRNA expression and several clinicopathological characteristics in the TCGA-STAD, GSE15459, and GSE62254 cohorts was analyzed. The results are shown in Fig. 2 and Tables S1 –S3. In the TCGA-STAD cohort, LBH expression was significantly higher in T3–T4 patients than in T1–T2 patients (*P* = 0.046). However, LBH expression was not associated with N and M stages (Fig. 2A). In the GSE15459 cohort, LBH was significantly associated with age (*P* = 0.020), Lauren classification (*P* = 0.019), stage (*P* = 0.017), and subtype (*P* < 0.001; Table S2). Specifically, the LBH expression in stages III–V was higher than in stages I–II (*P* = 0.0017, Fig. 2D). The invasive subtype has higher LBH expression than the proliferative and metabolic subtypes (*P* < 0.001, Fig. 2E). LBH expression in diffuse type was higher than in intestinal type (*P* = 0.022, Fig. 2F). In the GSE62254 cohort, LBH expression was higher in late T stage (T3–T4), N stage (N2–N3), and M stage (M1) (Fig. 2C). These results indicate that tumor proliferation and invasion is associated with LBH expression.

### LBH mRNA expression as independent predictor of OS in TCGA-STAD, GSE15459, and GSE62254 cohorts

Univariate Cox regression model showed that LBH was a significant predictor of OS in the TCGA-STAD cohort (*P* = 0.013). Other clinicopathological factors, including age (*P* = 0.021) and TNM stage (*P* < 0.001) were also found to be high-risk factors for OS (Table 1). Moreover, multivariate Cox regression analysis with variable selection showed that LBH (hazard ratio, HR = 1.487; 95% confidence interval, 95% CI [1.051–2.103]; *P* = 0.025), age (HR = 1.824, 95% CI [1.232–2.700]; *P* = 0.003), and TNM stage (HR = 1.598; 95% CI [1.288–1.969]; *P* < 0.001) were significant independent prognostic factors in GC (Table 1).

**Table 1:** Univariate and multivariate OS Analysis in TCGA-STAD cohort.

Variables	Univariate analysis			Multivariate analysis		
	HR	95%CI	*P* value	HR	95%CI	*P* value
LBH expression	1.529	1.094–2.138	0.013	1.487	1.051–2.103	0.025
Age(years)(<60 vs. ≥60)	1.556	1.070–2.263	0.021	1.824	1.232–2.700	0.003
Gender(Male vs. Female)	0.792	0.557–1.128	0.196			
TNM stage	1.541	1.254–1.895	<0.001	1.598	1.288–1.969	<0.001
Histological grade	1.325	0.962–1.826	0.085			

**Notes.**

Abbreviations: 95% CI 95% confidence interval OS overall survival T tumor invasion N lymph node M metastasis

The same method was applied in the GSE15459 and GSE62254 cohorts. In the GSE15459 cohort, univariate Cox model demonstrated that OS was significantly correlated with LBH expression (*P* < 0.001) and stage (*P* < 0.001). Multivariate Cox regression analysis revealed that LBH expression level was an independent prognostic factor in patients with GC (HR = 1.749; 95% CI [1.127–2.715]; *P* = 0.013; Table 2). Similarly, in the GSE62254 cohort, univariate Cox model demonstrated that OS was significantly correlated with LBH expression (*P* < 0.001), T stage (*P* < 0.001), N stage (*P* < 0.001), and M stage (*P* < 0.001). Furthermore, multivariate Cox regression analysis revealed that LBH expression level was an independent prognostic factor in patients with GC (HR = 1.450; 95% CI [1.015–2.072]; *P* = 0.041; Table 3).

**Table 2:** Univariate and multivariate OS Analysis in GSE15459 cohort.

Variables	Univariate analysis			Multivariate analysis		
	HR	95%CI	*P* value	HR	95%CI	*P* value
LBH expression	2.305	1.493–3.559	<0.001	1.749	1.127–2.715	0.013
Age (years)(<60 vs. ≥60)	0.983	0.641–1.506	0.936			
Gender (Male vs. Female)	0.713	0.462–1.101	0.127			
Lauren classification	0.844	0.611–1.166	0.302			
Stage	2.789	2.140–3.635	<0.001	2.789	2.140–3.635	<0.001
Subtype	0.967	0.797–1.174	0.736			

**Notes.**

Abbreviations: 95% CI 95% confidence interval OS overall survival

**Table 3:** Univariate and multivariate OS Analysis in GSE62254 cohort.

Variables	Univariate analysis			Multivariate analysis		
	HR	95%CI	*P* value	HR	95%CI	*P* value
LBH expression	1.862	1.316–2.634	<0.001	1.450	1.015–2.072	0.041
Age (years)(<60 vs. ≥60)	1.145	0.808–1.621	0.446			
Gender (Male vs. Female)	1.216	0.856–1.727	0.275			
T stage	1.871	1.486–2.356	<0.001	1.384	1.079–1.774	0.010
N stage	2.084	1.719–2.525	<0.001	1.810	1.479–2.214	<0.001
M stage	3.829	2.428–6.039	<0.001	2.209	1.380–3.534	0.001

**Notes.**

Abbreviations: 95% CI 95% confidence interval OS overall survival T tumor invasion N lymph node M metastasis

### LBH expression was elevated in GC tissues

**Figure 3:**
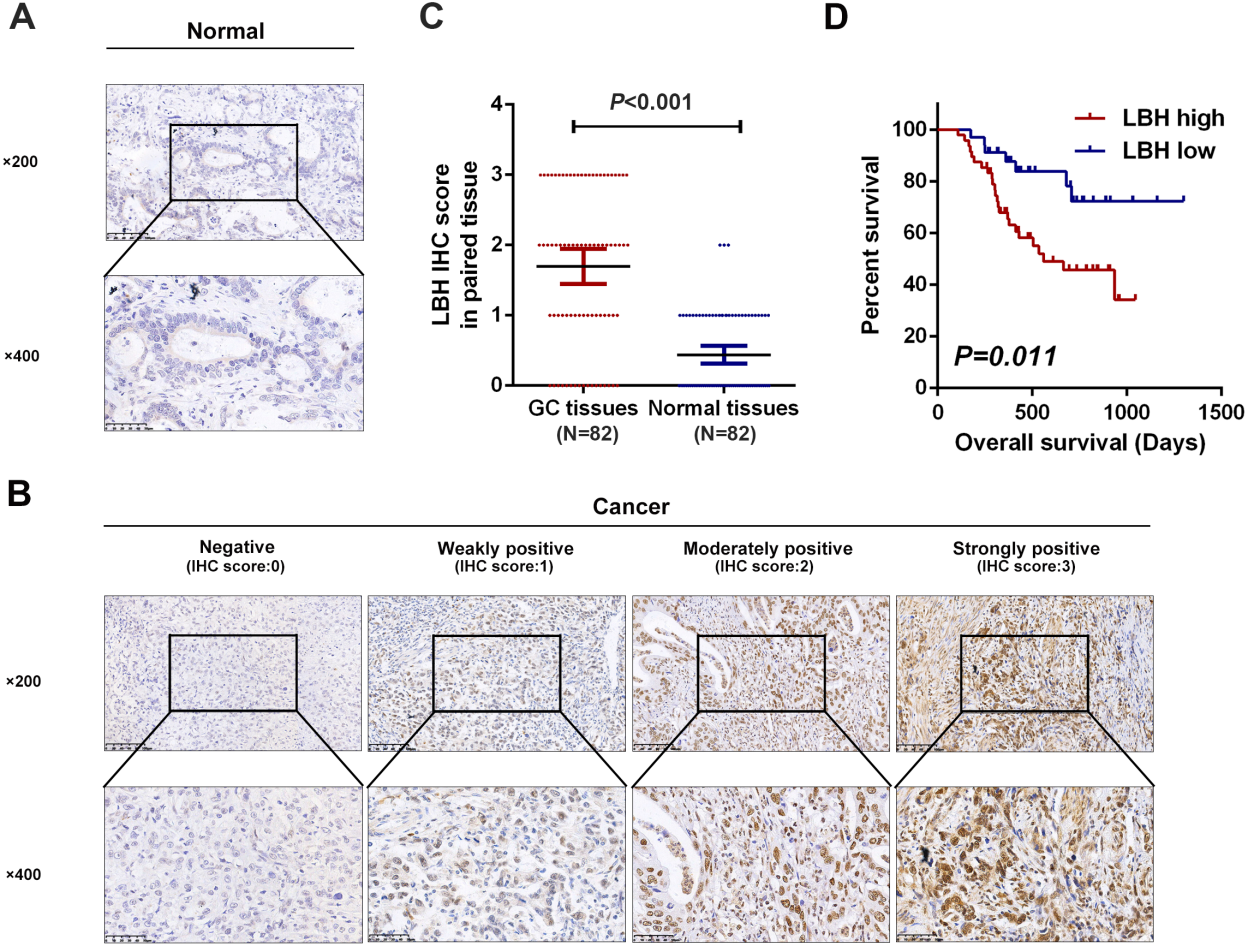
Detection of LBH protein expression in tissues by IHC staining. (A) Representative images of LBH protein expression in sections of non-neoplastic mucosa adjacent to tumors. The scales bars indicate 50 µm (upper) and 20 µm (lower). (B) IHC staining of LBH protein in GC tissues. IHC scoring was performed according to the staining intensity (0, negative; 1, weak; 2, moderated; 3, strong). The scales bars indicate 50 µm (upper) and 20µm (lower). (C) LBH protein expression was significantly increased in primary tumor specimens compared with adjacent non-tumor tissues by IHC (*P* < 0.001, *n* = 82). Each red dot represents an immunohistochemical score for a GC sample, and each blue dot represents an immunohistochemical score for a normal tissue. The middle horizontal line in the scatter dot plot represents the mean. Error bars represent mean ± s.d. Statistical significance (*P*) is indicated. (D) Kaplan-Meier analysis of LBH in GC patients. Patients with high LBH expression had shorter OS compared with low LBH expression (*P* = 0.011).

To validate the possible role of LBH in the development and progression of GC, the expression pattern of LBH protein was explored in paired clinical tissue samples in our own patient samples. Next, 82 pairs of GC and paracancerous normal tissues with complete clinical pathological parameters and follow-up information were respectively collected. LBH protein was predominantly distributed in the nucleus in GC tissues (Fig. 3B). The protein level of LBH was significantly higher in GC tissues than normal tissues (*P* <0.001, Figs. 3A,  3C). Taken together, these results confirmed that LBH was highly expressed in GC.

### Correlation between LBH expression and clinicopathological parameters in patients with GC

The association between LBH expression and clinicopathological parameters in patients with GC was further evaluated. As shown in Table 4, LBH expression in GC correlated with T stage (*P* = 0.046), N stage (*P* = 0.026), and stage (*P* < 0.001). Significant correlation was not found between LBH and age, gender, or differentiation grade. These results confirmed that LBH expression was associated with a malignant phenotype of GC.

**Table 4:** Clinicopathologic features of the patients in GC patients.

Characteristics	*N* = 82	LBH expression level			
		Low[n(%)]	High[n(%)]	*χ*2	*P* value
Gender				0.023	0.881
Male	61	25(41.0)	36(59.0)		
Female	21	9(42.9)	12(57.1)		
Age (year)				0	1.000
≤60	41	17(41.5)	24(58.5)		
>60	41	17(41.5)	24(58.5)		
T stage				3.979	0.046
T1-2	24	14(58.3)	10(41.7)		
T3-4	58	20(34.5)	38(65.5)		
N stage				4.952	0.026
N0-N1	46	24(52.2)	22(47.8)		
N2-N4	36	10(27.8)	26(72.2)		
Differentiation grade				2.751	0.097
Well	19	11(57.9)	8(42.1)		
Poor	63	23(36.5)	40(63.5)		
Stage				12.349	<0.001
I-II	39	24(61.5)	15(38.5)		
III	43	10(23.3)	33(76.7)		

**Notes.**

Notes T tumor invasion N lymph node

### Overexpression of LBH predicts poor prognosis in patients with GC

Next, the prognostic role of LBH was confirmed in our samples. Based on LBH protein expression levels, GC patients with complete follow-up information were divided (*N* = 82) into LBH low expression group (negative or weakly positive expression, *N* = 34) and LBH high expression group (moderately or strongly positive expression, *N* = 48). Kaplan-Meier curves and log-rank test analysis confirmed that patients with high LBH expression had significantly shorter OS than patients with low LBH expression (*P* = 0.011, Fig. 3D).

### LBH serves as an independent prognostic marker in GC patients

To validate the potential of LBH as an independent prognostic factor in GC patients, Cox regression analysis was used to examine the OS. Univariate analysis indicated that LBH expression (HR = 2.853, 95% CI [1.228–6.629], *P* = 0.015), N stage (HR = 1.830, 95% CI [1.374–2.436], *P* < 0.001), and differentiation grade (HR = 3.253, 95% CI [1.131–9.356], *P* = 0.029) were associated with OS. Furthermore, multivariate analysis demonstrated that LBH expression (HR = 2.371, 95% CI [1.012–5.555], *P* = 0.047) and N stage (HR = 1.766, 95% CI [1.322–2.359], *P* <0.001) were independent prognostic factors for OS (Table 5). Taken together, LBH can serve as an independent prognostic factor in patients with GC.

**Table 5:** Univariate and multivariate OS Analysis in GC patients.

Variables	Univariate analysis			Multivariate analysis		
	HR	95%CI	*P* value	HR	95%CI	*P* value
LBH expression	2.853	1.228–6.629	0.015	2.371	1.012–5.555	0.047
Age (years)(<60 vs. ≥60)	0.998	0.492–2.027	0.996			
Gender (Male vs. Female)	1.229	0.565–2.674	0.603			
T stage	1.395	0.971–2.004	0.072			
N stage	1.830	1.374–2.436	<0.001	1.766	1.322–2.359	<0.001
Differentiation grade (poor vs. well)	3.253	1.131–9.356	0.029			

**Notes.**

Abbreviations: 95% CI 95% confidence interval OS overall survival T tumor invasion N lymph node

### LBH overexpression promoted cell proliferation and invasion in BGC-823 cells

**Figure 4:**
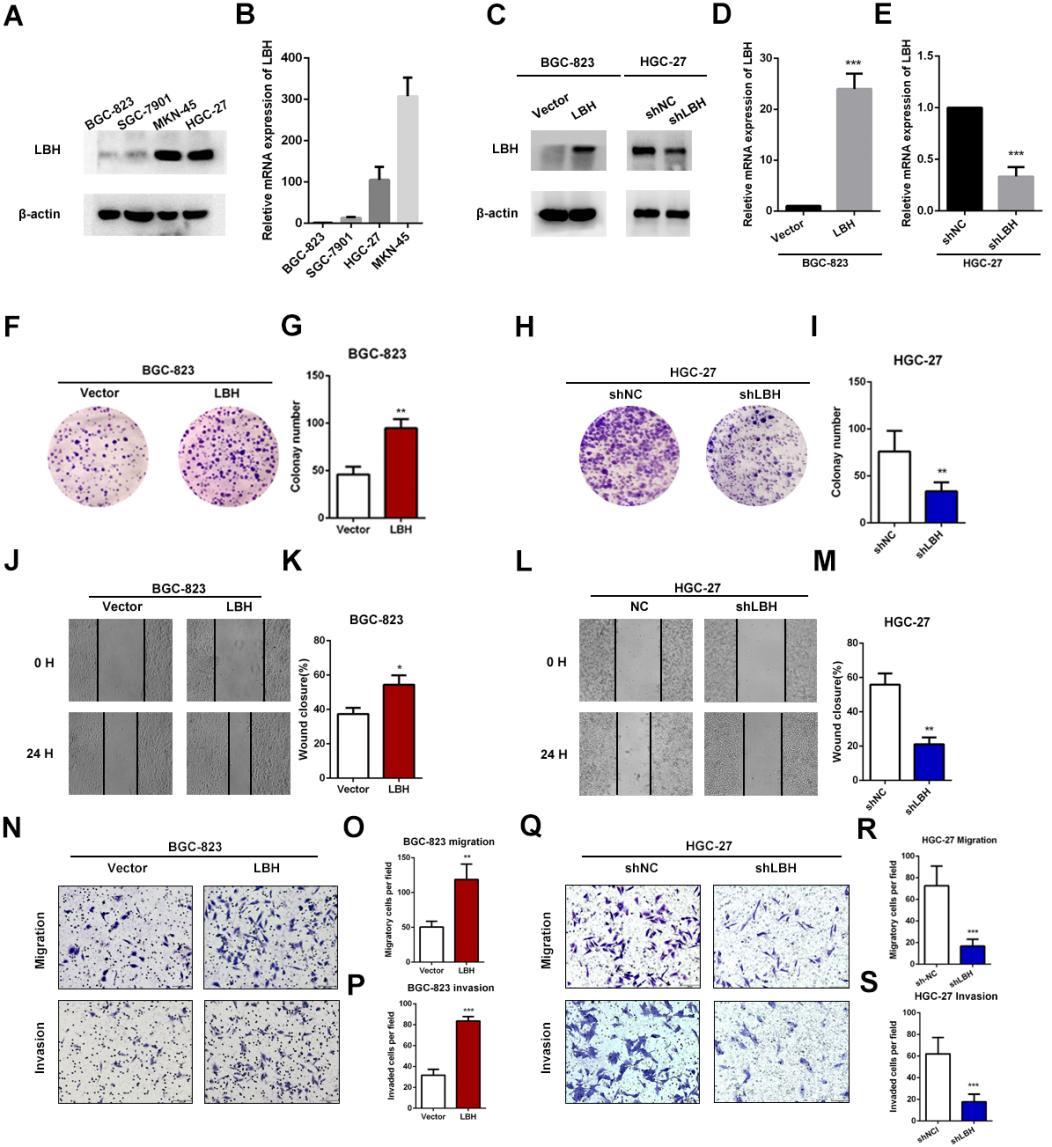
LBH promoted the gastric cancer cell proliferation, migration and invasion. (A) Protein expression of LBH in gastric cancer cell lines by western blot. (B) LBH mRNA expression in gastric cancer cell lines by qRT-PCR. (C–E) Lentivirus was used for overexpression or knockdown of LBH, and the efficiency was measured by western blot (C) and qRT-PCR (D–E). *** *P* < 0.001, based on Student’s t-test. (F–G) Colony numbers of BGC-823 cells stably overexpressing LBH were more than those transfected with vector. ** *P* < 0.01, based on Student’s t-test. (H–I) Colony numbers of HGC-27 cells transfected with LBH-shRNA were less than those transfected with NC-shRNA. ** *P* < 0.01, based on Student’s t-test. (J–K) BGC-823 cells were transfected with LBH and vector lentivirus, subsequently analyzed with scratch wound healing assay. * *P* < 0.05, based on Student’s t-test. (L–M) Wound healing assay for the evaluation of LBH knockdown on HGC-27 migration ability. ** *P* < 0.01, based on Student’s t-test. (N–P) BGC-823 cells were transfected with LBH and vector lentivirus, subsequently analyzed with transwell migration and invasion assay. ** *P* < 0.01, *** *P* < 0.001, based on Student’s t-test. (Q–S) HGC-27 cells were transfected with NC-shRNA and LBH-shRNA lentivirus, subsequently analyzed with transwell migration and invasion assay. *** *P* < 0.001, based on Student’s t-test. Each set of experiments was repeated three times.

The expression levels of LBH were compared in four GC cell lines. The results showed that BGC-823 cells had the lowest LBH expression among the four tested GC cell lines, and HGC-27 and MKN-45 had higher LBH expression (Figs. 4A–4B). BGC-823 cells were transfected with LBH overexpression lentivirus and vector. LBH mRNA and protein expression levels were significantly increased in BGC-823 cells transfected with LBH overexpression lentivirus compared with vector (Figs. 4C–4D). Colony formation experiments showed the colony number of LBH overexpression BGC-823 cells was significantly higher than vector BGC-823 cells (*P* = 0.0026, Figs. 4F–4G). Wound healing experiments showed that LBH overexpression significantly increased the wound healing capability of BGC-823 cells (*P* = 0.0108, Figs. 4J–4K). Transwell migration and Matrigel invasion assays showed that LBH overexpression promoted migration and invasion of BGC-823 cells with statistically significant difference (*P* = 0.0076 and *P* = 0.0002, respectively, Figs. 4N–4P). These findings indicated that overexpression of LBH can promote the proliferation and invasion of GC cells.

### LBH knockdown inhibited cell proliferation and invasion in HGC-27 cells

To verify the function of LBH in HGC-27 cells, knockdown experiments using short hairpin RNAs (shRNAs) were performed (Figs. 4C, 4E). LBH knockdown significantly suppressed cell proliferation based on colony formation experiments (Figs. 4H–4I). The migration abilities of HGC-27 cells in the shLBH-transfected group showed significant decline after being wounded for 24 h (Figs. 4L–4M) or being passed through the polycarbonate membrane for 24 h (Figs. 4Q–4R). Furthermore, the Transwell chamber (with Matrigel) assay showed the invasive potential of HGC-27 cells was significantly weakened in the shLBH-transfected group (Fig. 4Q, Fig. 4S).

### Gene set enrichment analysis (GSEA) in three independent datasets

**Figure 5:**
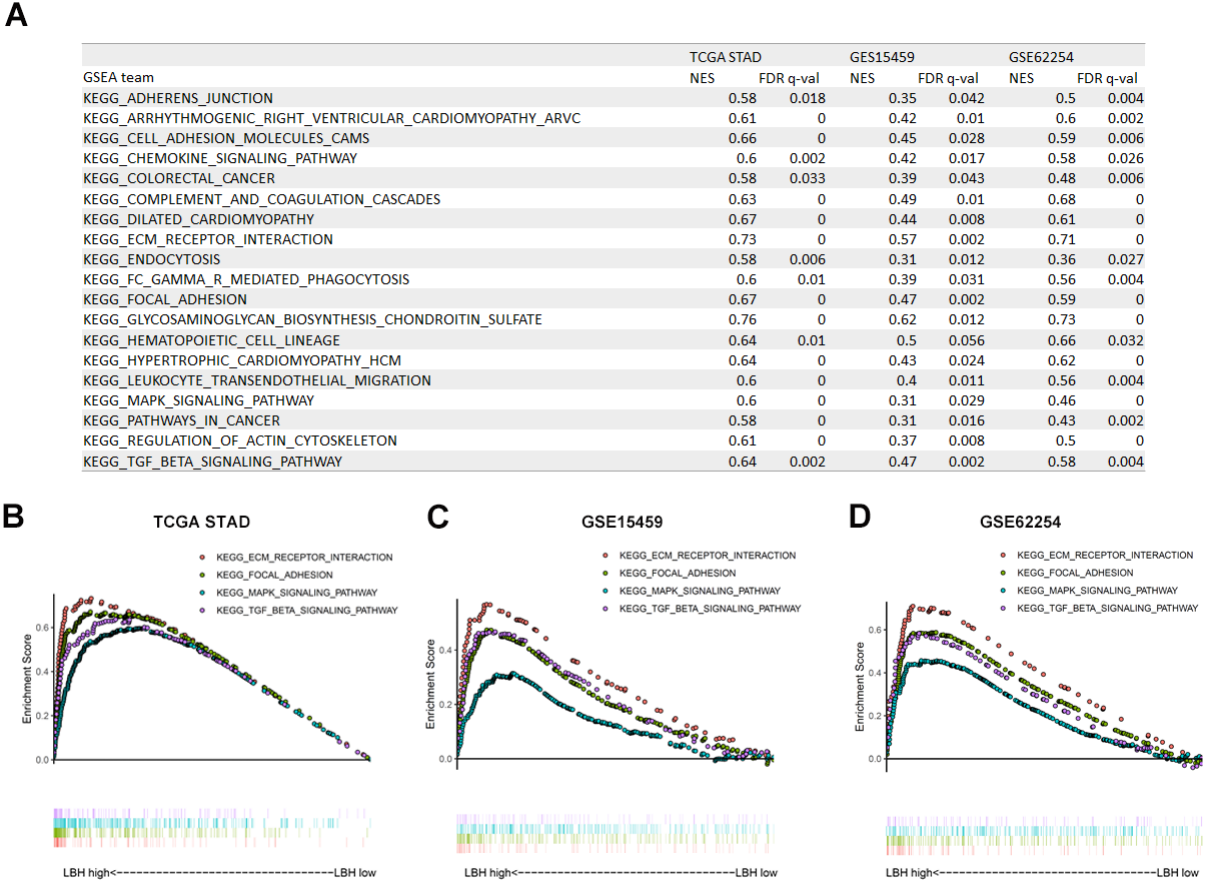
Gene Set Enrichment Analysis (GSEA) result of LBH in three independent datasets. (A) GSEA terms that are significantly enriched in TCGA-STAD, GSE15459 and GSE62254. (B–D) KEGG pathway, named “KEGG_ECM_RECPTOR_INTERACTION”, “KEGG_FOCAL_ADHENSION”, “KEGG_MAPK_SIGNAL_PATHWAY” and “KEGG_TGF_BETA_SIGNAL_PATHWAY” was enriched in TCGA-STAD(B), GSE15459(C) and GSE62254(D) cohort. The position of the color bars indicates the ordering of the differential genes relative to other genes. The colored dots indicate the strength of the enriched genes under high LBH conditions (left) or low LBH conditions (right).

To investigate how LBH affects the prognosis of GC patients, GSEA was performed in TCGA-STAD, GSE15459, and GSE62254 cohorts. Using a FDR <0.05 as standard, 76, 22, and 40 pathways in TCGA-STAD, GSE15459, and GSE62254 cohorts, respectively, were enriched to correlate with high LBH expression. Of these, 21 pathways were enriched in all three datasets (Fig. 5A). Important pathways in the development and progression of tumors, such as ECM-receptor interaction, focal adhesion, the MAPK signal pathway, and the TGF- β signal pathway were enriched in all three data sets (Figs. 5B–5D). This result indicates that LBH may participate in the development of cancer by regulating cancer adhesion process or participating in cancer pathways.

### GO and KEGG analyses of LBH co-expressed genes

**Figure 6:**
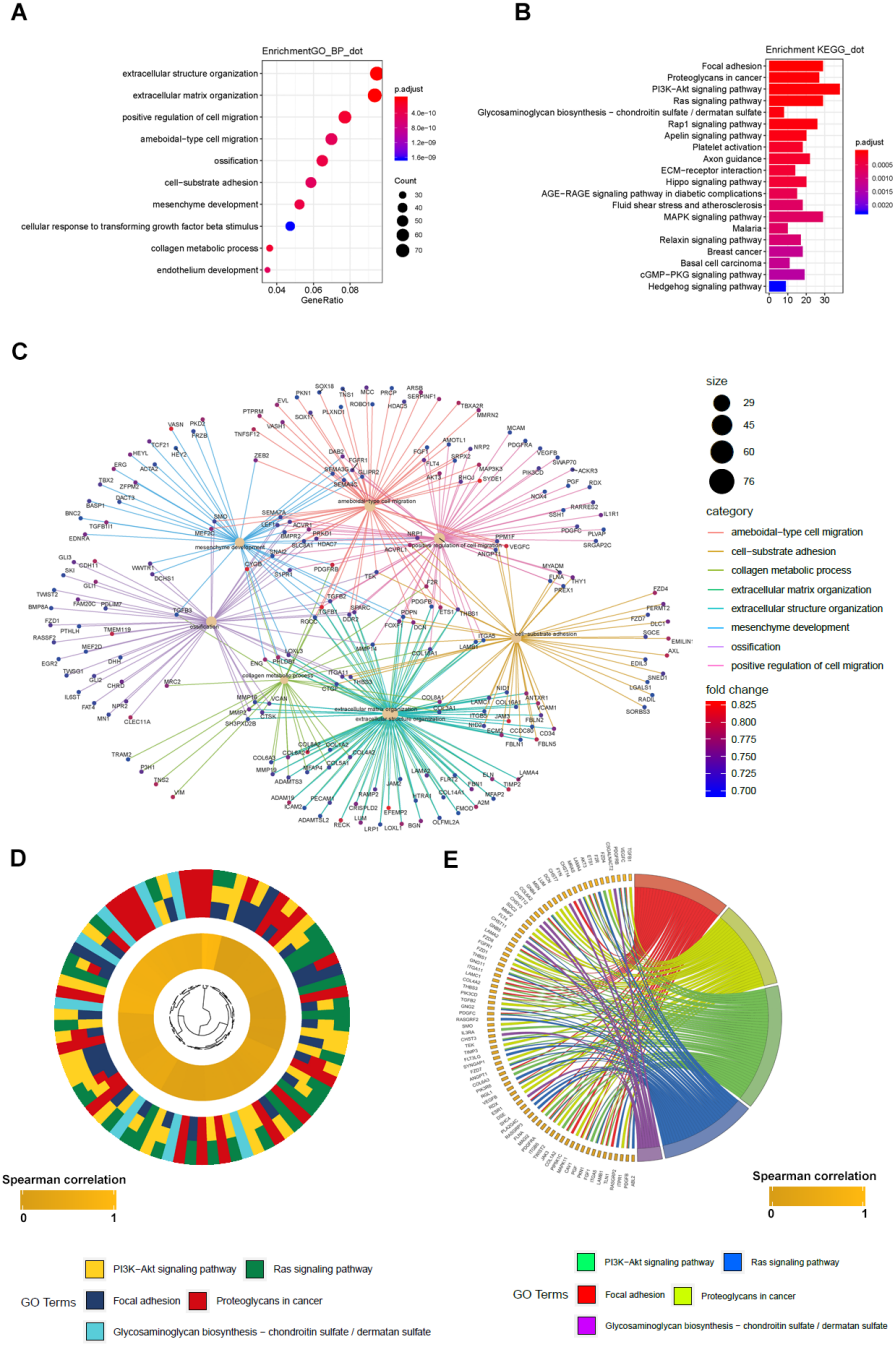
GO_BP and KEGG result of LBH co-expression gene in TCGA STAD. (A) LBH co-expression gene in TCGA were enriched in biological process related to cell migration and extracellular matrix. Fold enrichment of each GO term are indicated by the *x*-axis and bar color. (B) LBH co-expression gene in TCGA were enriched in KEGG pathways cell proliferation, migration and adhesion. Fold enrichment of each KEGG term are indicated by the *x*-axis and bar color. (C) GO-BP analysis for the co-expression genes of LBH. The brown node represents the enriched GO-BP term, with the size indicating the overall number of its included genes. The other smaller nodes are the enriched mRNAs, and the node colors changing from green to red indicate the increased associations of the mRNAs with LBH. (D) Hierarchical clustering of the LBH co-expression genes profiles in each KEGG pathways. (E) Chord plot displays of the relationship between genes and KEGG pathways. Spearman correlation coefficient of gene expression were indicated as colored squares.

To further elucidate the molecular mechanism of LBH, we performed GO and KEGG analyses on LBH co-expressed genes in the TCGA-STAD cohort. GO analysis revealed the top 1,000 genes co-expressed with LBH were mainly enriched in biological processes associated with ECM, cell movement and cell adhesion (Fig. 6A). KEGG analysis showed that LBH co-expressed genes were enriched in terms of local adhesion, proteoglycans in cancer, and participates in classical cancer pathways such as PI3K-Akt, Ras, and Rap1 (Fig. 6B). The genes specifically enriched in each of the GO and KEGG terms are shown in Figs. 6C–6E. Therefore, the results from this study indicate that LBH may activate a range of cancer-associated pathways and lead to tumor proliferation and invasion phenotypes. This is consistent with the results from the correlation analysis of LBH and clinicopathological parameters described above.

### LBH is involved in the Integrin/FAK/Akt signaling pathway

**Figure 7:**
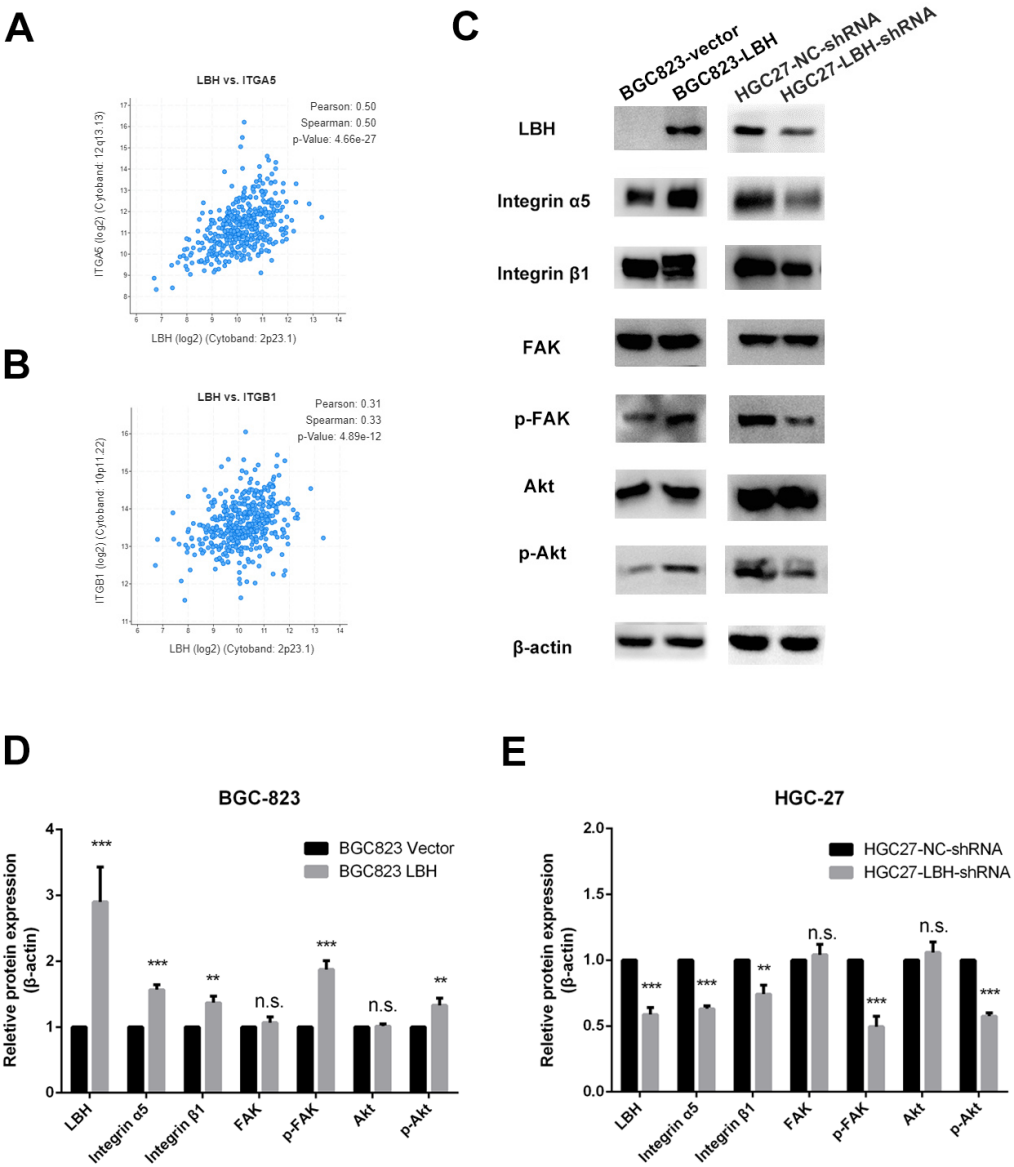
The effect of LBH on integrin α5 β1/FAK/Akt pathway. (A) The expression of LBH (x) and ITGA5 (y) in GC. Analysis was performed with cBioPortal on the provisional TCGA datasets. (B) The expression of LBH (x) and ITGB1 (y) in GC. Analysis was performed with cBioPortal on the provisional TCGA datasets. (C) The effect of LBH overexpression or knockdown on protein level of key Integrin/FAK/Akt pathway markers by western blot. Each experiment was repeated three times. (D–E) Actin bands represent equivalence in protein loading. The optical densities of each protein band were measured using ImageJ software. Student’s t-test was used for statistical analysis. Data are expressed as mean ±s.d. Differences are considered significant at * *P* < 0.05; ** *P* < 0.01; *** *P* < 0.001.

The results of GSEA and KEGG indicate that LBH is associated with focal adhesion, ECM-receptor interaction, and PI3K/Akt signaling pathways. Integrin extensively mediates the process of cell adhesion and can activate the PI3K/Akt signaling pathway. In addition, the pathway is widely involved in tumor proliferation and metastasis. Analysis of LBH co-expressed genes in the TCGA-STAD cohort showed a positive correlation between LBH and ITGA5 and ITGB1 (Figs. 7A–7B). Western blot results confirmed that knockdown of LBH in HGC-27 cells significantly reduced the expression of integrin α5 and β1 compared with the control group (Figs. 7C–7E). FAK is one of the main downstream molecules of integrin α5β1. As shown in Fig. 7C, after LBH knockdown, the p-FAK level was significantly decreased compared with the control group, however, FAK was not significantly changed. Integrin/FAK can further activate the PI3K/Akt signaling pathway. Western blot analysis showed that after LBH knockdown, p-Akt levels decreased significantly although Akt did not change significantly. To further validate the results, LBH was overexpressed in BGC-823 cells. The results showed that after LBH overexpression, the integrin α5β1 was significantly upregulated. Furthermore, the levels of p-FAK and p-Akt were significantly upregulated after overexpression of LBH compared with the control group. These results indicated that LBH can upregulate integrin α5β1, thereby activating the integrin/FAK/Akt signaling pathway, leading to cell proliferation and invasion.

## Discussion

GC is a highly malignant tumor. Once metastasis occurs, more than half of patients die within one year (Shitara et al., 2018). Therefore, the search for prognostic markers of GC is an area of current research interest (Zhang et al., 2015a). In this study, the expression of LBH was confirmed using online datasets and immunohistochemical specimens to predict the prognosis of patients. The OS in the LBH high expression group was extended significantly compared with the LBH low expression group. Simultaneously, the effect of LBH on prognosis was independent of TNM staging, which further confirms the importance of LBH.

Novel prognostic markers have been investigated in many previous studies. However, few of the research conclusions can be extended to the clinical setting. The likely reason is the number of sample cases in those studies limits the reliability of the conclusions. Large sample RNA-seq studies provide a good solution to this problem. In the present study, to increase the credibility of the results, the TCGA-STAD, GSE15459, GSE62254, and GSE29272 datasets were selected. Since these datasets are derived from previous studies, they have different baseline characteristics of the patients included (Tables S1–S3). In addition, LBH is a relatively newly discovered molecule, and the impact of various baseline characteristics is difficult to predict, thus, in the present study, multiple verifications were performed in several datasets to obtain a more credible conclusion. Consequently, in multiple independent datasets, the prognosis of patients could be determined based on LBH expression. Studies based on the public datasets are at the RNA level, however, RNA needs to be translated into proteins to function. Therefore, the results were verified using immunohistochemistry in our sample of patients, and the prognostic efficacy of LBH was confirmed.

Based on the analysis of public datasets and immunohistochemistry results, LBH was significantly correlated with T and N stages (Fig. 2), which was verified with *in vitro* experiments. Results showed overexpression of LBH significantly promoted the proliferation and invasion of GC cells, while knockdown of LBH significantly inhibited the proliferation and invasion of GC cells. The proliferation and migration of LBH have been investigated in many previous studies. Liu found that LBH inhibits the proliferation of CNE1 cells in nasopharyngeal carcinoma by causing G1/S phase arrest (Liu et al., 2015). In breast cancer, LBH is considered an oncogene directly regulated by the Wnt/ β-catenin pathway, and LBH overexpression leads to a more aggressive basal differentiation of breast cancer (Lindley et al., 2015). Due to the heterogeneity of cancers, different pathways are involved in proliferation and metastasis in various types of cancer, which may be the reason for the diversity of LBH action.

In an attempt to explain the action mechanism of LBH, GSEA was first performed with the online datasets TCGA-STAD, GSE15459, and GSE62254 using the same parameter settings. Notably, the results enriched in the three datasets were highly reproducible (Fig. 5), involving pathways between the ECM and the adhesion process, such as focal adhesion and ECM-receptor interaction in the three GSEAs. To further validate the results, the TCGA-STAD dataset was used for LBH co-expression gene screening and KEGG analysis. The analysis used a hypergeometric test approach that was different from the GSEA approach to discover a possible mechanism for the role of LBH from another perspective. Similar to GSEA, adhesion-related pathways, such as ECM-receptor interaction, and signaling pathways associated with proliferation and metastasis, PI3K-Akt and MAPK, were screened to provide a possible explanation for the results of *in vitro* studies.

Due to the strong correlation between LBH and ITGB1 and ITGA5 in the TCGA-STAD datasets, combined with the results of GSEA and KEGG, we hypothesized that LBH may activate integrin/FAK pathway by increasing the expression of integrin, thereby activating the downstream Akt pathway. Activation leads to enhanced cell proliferation and migration. Western blot analysis showed that integrin α5β1 was significantly enhanced after overexpression of LBH. FN binds to integrin α5β1 and promotes phosphorylation of FAK, leading to tumor proliferation and metastasis (Sulzmaier, Jean & Schlaepfer, 2014). The increase in p-FAK and p-Akt was observed after overexpression of LBH, consistent with our hypothesis. LBH acts as a transcription cofactor primarily in the nucleus, which can bind to the AP-1 transcription factor, resulting in enhanced transcriptional activity of AP-1 (Ai et al., 2008). Integrin β1 is predicted to be regulated by AP-1 (Table S8), explaining why LBH causes an increase in integrin to some extent.

Based on the above experimental results, the LBH expression level in GC, and its relationship with clinicopathological parameters and prognosis of GC patients, showed the potential clinical value of LBH. This is the first study in which LBH was shown to promote the upregulation of integrin α5β1 in GC cells, thereby activating the integrin/FAK/Akt signaling pathway, and in turn, promoting the proliferation and invasion of GC, indicating that LBH may be an important cancer-promoting factor in GC. The present study had several limitations for explaining the mechanism. First, the bioinformatics functional analysis of LBH in the online datasets does not fully reveal the function of LBH because the LBH level may be regulated by other factors. Sequencing after overexpression or knockdown of LBH in our cell line could help solve this problem. Second, the specific mechanism by which LBH regulates integrin α5β1 has not been elucidated fully. Further study *in vitro* and *in vivo* is needed.

## Conclusion

In conclusion, results from the present study showed that LBH is overexpressed in GC and high LBH expression indicated a shorter OS in GC patients. By upregulating integrin α5β1 in GC cells, LBH can activate the integrin/FAK/Akt signaling pathway, thereby promoting the proliferation and invasion of GC. The study results indicate that LBH may serve as a new prognostic biomarker and potential therapeutic target for GC.

##  Supplemental Information

10.7717/peerj.6885/supp-1 Table S1 Clinicopathologic features of the patients inTCGA- This table shows the relationship between LBH expression levels and clinical pathological parameters in 375 GC patients in the TCGA-STAD dataset. Patients were divided into high and low groups based on the median of LBH expression values in this dataset. Chi-square test is used for statistics. Abbreviations: T, tumor size; N, lymph node; M stage, metastasis.

10.7717/peerj.6885/supp-2 Table S2 Clinicopathologic features of the patients in GSE15459 This table shows the relationship between LBH expression levels and clinical pathological parameters in 192 GC patients in the GSE15459 data set. Patients were divided into high and low groups based on the median of LBH expression values in this dataset. Chi-square test is used for statistics.

10.7717/peerj.6885/supp-3 Table S3 Clinicopathologic features of the patients in GSE62254 This table shows the relationship between LBH expression levels and clinical pathological parameters in 300 GC patients in the GSE62254 dataset. Patients were divided into high and low groups based on the median of LBH expression values in this dataset. Chi-square test is used for statistics. Abbreviations: T, tumor size; N, lymph node; M stage, metastasis.

10.7717/peerj.6885/supp-4 Table S4 LBH expression in cancer and cancer in TCGA STAD Expression values of LBH in GC and normal gastric tissues in the TCGA-STAD data set. The data is in FPKM format. “01” represents a gastric cancer sample, and ”11” represents a normal gastric tissue sample.

10.7717/peerj.6885/supp-5 Table S5 Expression of LBH in cancer tissues and adjacent normal tissues in GSE29272 dataset Expression values of LBH in GC and adjacent tissues in GSE29272 dataset. ”1” represents a tissue sample of gastric cancer, and ”0” represents a sample of adjacent tissues.

10.7717/peerj.6885/supp-6 Table S6 LBH co-expression gene in TCGA STAD 1000 genes with the highest spearman correlation coefficient with LBH in TCGA-STAD dataset.

10.7717/peerj.6885/supp-7 Table S7 Raw data for statistical analysis The statistical analysis in Figs. 4 and  7 is listed in the table below. Each experiment was performed three times independently.

10.7717/peerj.6885/supp-8 Table S8 The target gene of c-jun predicted by CHIP-seq The data was downloaded from GRTD ( www://gtrd.biouml.org). The CHIP-seq data showed that c-jun has 61 binding sites with the ITGB1 promoter region.

10.7717/peerj.6885/supp-9 Dataset S1 Raw data of TCGA STAD, GSE15459, GSE62254 and 82 patients with gastric cancer The .sav data is the SPSS format. Each individual .sav data contains LBH expression, LBH grouping, and clinical pathology parameters of the patient.

10.7717/peerj.6885/supp-10 Dataset S2 Raw data of western blot in Fig. 4 Each image represents an uncut western sub-strip, and the name of the image is the primary antibody used. The irrelevant strips are painted off in black.

10.7717/peerj.6885/supp-11 Dataset S3 Raw data of western blot in Fig. 7 Each image represents an uncut western sub-strip, and the name of the image is the primary antibody used. The irrelevant strips are painted off in black.

10.7717/peerj.6885/supp-12 Dataset S4 Dataset S4 The raw data of GSEA in TCGA-STAD, GSE15459 and GSE62254 datasets.

## Additional Information and Declarations

### Data Availability

The following information was supplied regarding data availability: Raw data is available in the Supplemental Files.
