# Supplementary material for: Elevated limb-bud and heart development (LBH) expression indicates poor prognosis and promotes gastric cancer cell proliferation and invasion via upregulating Integrin/FAK/Akt pathway
Source: PeerJ. 2019 May 6;7:e6885. doi: 10.7717/peerj.6885 (PMC6507893; doi:10.7717/peerj.6885)
Supplement: Table S1 — This table shows the relationship between LBH expression levels and clinical pathological parameters in 375 GC patients in the TCGA-STAD dataset. Patients were divided into high and low groups based on the median of LBH expression values in this dataset. Chi-square test is used for statistics. Abbreviations: T, tumor size; N, lymph node; M stage, metastasis. [file peerj-07-6885-s001.docx]

| Characteristics | N=375 | LBH expression level | |  |  |
| --- | --- | --- | --- | --- | --- |
|  |  | Low[n(%)] | High[n(%)] | χ2 | *P* value |
| Gender |  |  |  | 0.370 | 0.543 |
| Male | 241 | 118(49.0%) | 123(51.0%) |  |  |
| Female | 134 | 70(52.2%) | 64(47.8%) |  |  |
| Age(year) |  |  |  | 1.564 | 0.211 |
| ≤60 | 121 | 55(45.5%) | 66(54.5%) |  |  |
| ＞60 | 254 | 133(52.4%) | 121(47.6%) |  |  |
| T stage |  |  |  | 12.737 | 0.005 |
| T1 | 19 | 17(89.5%) | 2(10.5%) |  |  |
| T2 | 80 | 40(50.0%) | 40(50.0%) |  |  |
| T3 | 168 | 85(50.6%) | 83(49.4%) |  |  |
| T4 | 100 | 45(45.0%) | 55(55.0%) |  |  |
| N stage |  |  |  | 4.693 | 0.196 |
| N0 | 111 | 64(54.7%) | 47(42.3%) |  |  |
| N1 | 97 | 50(51.5%) | 47(48.5%) |  |  |
| N2 | 75 | 36(48.0%) | 39(52.0%) |  |  |
| N3 | 74 | 31(41.9%) | 43(58.1%) |  |  |
| M stage |  |  |  | 0.011 | 0.916 |
| M0 | 330 | 168(50.9%) | 162(49.1%) |  |  |
| M1 | 25 | 13(52.0%) | 12(48.0%) |  |  |
| TNM stage |  |  |  | 10.194 | 0.017 |
| I | 53 | 37(69.8%) | 16(30.2%) |  |  |
| II | 111 | 49(44.1%) | 62(55.9%) |  |  |
| III | 150 | 73(48.7%) | 77(51.3%) |  |  |
| IV | 38 | 21(55.3%) | 17(44.7%) |  |  |
| Histological grade |  |  |  | 2.771 | 0.250 |
| G1 | 10 | 6(60.0%) | 4(40.0%) |  |  |
| G2 | 137 | 76(55.5%) | 61(44.5%) |  |  |
| G3 | 219 | 103(47.0%) | 116(53.0%) |  |  |

**Table S1** Clinicopathologic features of the patients in TCGA STAD
