# Supplementary material for: Elevated limb-bud and heart development (LBH) expression indicates poor prognosis and promotes gastric cancer cell proliferation and invasion via upregulating Integrin/FAK/Akt pathway
Source: PeerJ. 2019 May 6;7:e6885. doi: 10.7717/peerj.6885 (PMC6507893; doi:10.7717/peerj.6885)
Supplement: Table S2 — This table shows the relationship between LBH expression levels and clinical pathological parameters in 192 GC patients in the GSE15459 data set. Patients were divided into high and low groups based on the median of LBH expression values in this dataset. Chi-square test is used for statistics. [file peerj-07-6885-s002.docx]

**Table S2** Clinicopathologic features of the patients in GSE15459 dataset

| Characteristics | N=192 | LBH expression level | |  |  |  |  |  |
| --- | --- | --- | --- | --- | --- | --- | --- | --- |
|  |  | Low[n(%)] | High[n(%)] | χ2 | *P* value |  |  |  |
| Gender |  |  |  | 0.023 | 0.880 |  |  |  |
| Male | 125 | 62(49.6) | 63(50.4) |  |  |  |  |  |
| Female | 67 | 34(50.7) | 33(49.3) |  |  |  |  |  |
| Age(year) |  |  |  | 5.505 | 0.019 |  |  |  |
| ≤60 | 117 | 22(37.3) | 37(62.7) |  |  |  |  |  |
| ＞60 | 183 | 74(55.6) | 59(44.4) |  |  |  |  |  |
| Lauren classification |  |  |  | 7.955 | 0.019 |  |  |  |
| Diffuse | 75 | 28(37.3) | 47(62.7) |  |  |  |  |  |
| Intestinal | 99 | 58(58.6) | 41(41.4) |  |  |  |  |  |
| Mixed | 18 | 10(55.6) | 8(44.4) |  |  |  |  |  |
| Stage |  |  |  | 10.197 | 0.017 |  |  |  |
| I | 31 | 22(71.0) | 9(29.0) |  |  |  |  |  |
| II | 29 | 18(62.1) | 11(37.9) |  |  |  |  |  |
| III | 72 | 31(43.1) | 41(56.9) |  |  |  |  |  |
| IV | 60 | 25(41.7) | 35(58.3) |  |  |  |  |  |
| Subtype |  |  |  | 34.272 | <0.001 |  |  |  |
| Invasive | 51 | 9(17.6) | 42(82.4) |  |  |  |  |  |
| Metabolic | 40 | 29(72.5) | 11(27.5) |  |  |  |  |  |
| Proliferative | 70 | 44(62.9) | 26(37.2) |  |  |  |  |  |
| Unstable | 31 | 14(45.2) | 17(54.8) |  |  |  |  |  |
