# Supplementary material for: Elevated limb-bud and heart development (LBH) expression indicates poor prognosis and promotes gastric cancer cell proliferation and invasion via upregulating Integrin/FAK/Akt pathway
Source: PeerJ. 2019 May 6;7:e6885. doi: 10.7717/peerj.6885 (PMC6507893; doi:10.7717/peerj.6885)
Supplement: Table S3 — This table shows the relationship between LBH expression levels and clinical pathological parameters in 300 GC patients in the GSE62254 dataset. Patients were divided into high and low groups based on the median of LBH expression values in this dataset. Chi-square test is used for statistics. Abbreviations: T, tumor size; N, lymph node; M stage, metastasis. [file peerj-07-6885-s003.docx]

**Table S3** Clinicopathologic features of the patients in GSE62254 dataset

| Characteristics | N=300 | LBH expression level | |  |  |
| --- | --- | --- | --- | --- | --- |
|  |  | Low[n(%)] | High[n(%)] | χ2 | *P* value |
| Gender |  |  |  | 23.163 | 0.038 |
| Male | 199 | 108(54.3) | 91(45.7) |  |  |
| Female | 101 | 42(41.6) | 59(58.4) |  |  |
| Age(year) |  |  |  | 7.412 | 0.006 |
| ≤60 | 117 | 47(40.2) | 70(59.8) |  |  |
| ＞60 | 183 | 103(56.3) | 80(43.7) |  |  |
| T stage |  |  |  | 23.163 | <0.001 |
| T1 | 0 | 0(0.0) | 0(0.0) |  |  |
| T2 | 188 | 114(60.6) | 74(39.4) |  |  |
| T3 | 91 | 28(30.8) | 63(69.2) |  |  |
| T4 | 21 | 8(38.1) | 13(61.9) |  |  |
| N stage |  |  |  | 6.088 | 0.107 |
| N0 | 38 | 24(63.2) | 14(36.8) |  |  |
| N1 | 131 | 70(53.4) | 61(46.6) |  |  |
| N2 | 80 | 35(43.8) | 45(56.3) |  |  |
| N3 | 51 | 21(41.2) | 30(58.8) |  |  |
| M stage |  |  |  | 4.925 | 0.026 |
| M0 | 273 | 142(52.0) | 131(48.0) |  |  |
| M1 | 27 | 8(29.6) | 19(70.4) |  |  |
| TNM stage |  |  |  | 19.637 | <0.001 |
| I | 30 | 21(70.0) | 9(30.0) |  |  |
| II | 97 | 61(62.9) | 36(37.1) |  |  |
| III | 96 | 40(41.7) | 56(58.3) |  |  |
| IV | 77 | 28(36.4) | 49(63.6) |  |  |
